# Ketamine as an NMDA-modulating therapy in bipolar disorder: rationale and evidence

**DOI:** 10.3389/fpsyt.2026.1777402

**Published:** 2026-03-04

**Authors:** R. Queissner, F. Fellendorf, E. Z. Reininghaus

**Affiliations:** Clinical Division for Psychiatry and Psychotherapeutic Medicine, Medical University of Graz, Graz, Austria

**Keywords:** bipolar depression, esketamine, ketamine, NMDA receptor, treatment-resistant depression (TRD)

## Abstract

**Background:**

Bipolar depression remains a leading cause of morbidity, functional impairment, and suicide risk in bipolar disorder. Conventional pharmacotherapies often provide delayed or incomplete relief and may increase the risk of treatment-emergent affective switching. Recent evidence highlights the therapeutic potential of NMDA receptor antagonists, particularly ketamine and its S-enantiomer esketamine, as rapid-acting antidepressants.

**Methods:**

This narrative review synthesizes findings from randomized controlled trials, systematic reviews, and real-world studies investigating ketamine and esketamine in treatment-resistant bipolar depression. Emphasis was placed on antidepressant efficacy, safety, and the risk of mood destabilization.

**Results:**

Across multiple trials, ketamine produced rapid and robust antidepressant effects, with significant improvement in depressive symptoms within hours of administration. Data confirmed high response rates in treatment-resistant bipolar depression, while rapid-onset benefits with minimal switch risk. Further there is evidence on comparable efficacy and safety of intranasal esketamine in bipolar and unipolar depression, with no cases of mania or hypomania.

**Conclusions:**

Ketamine and esketamine represent mechanistically novel and clinically effective treatments for bipolar depression. Their rapid antidepressant action and low switch liability distinguish them from traditional antidepressants. Esketamine, with its favorable tolerability and intranasal administration, offers a promising adjunctive option for treatment-resistant bipolar depression. Long-term data are warranted to optimize maintenance strategies and confirm sustained safety and efficacy.

## Introduction

Bipolar disorder (BD) is a chronic, recurrent psychiatric illness characterized by alternating episodes of depression, mania, or mixed affective states, often resulting in profound functional impairment and elevated suicide risk. Among its clinical phases, bipolar depression represents the most prevalent and therapeutically challenging state, particularly in treatment-resistant bipolar depression (TRBD). Conventional pharmacological interventions—including mood stabilizers, second-generation antipsychotics, and selective antidepressants—frequently produce incomplete responses, delayed onset of action, or undesired affective switching, limiting their clinical utility (1).

Neurobiological and neuroimaging research increasingly supports the role of glutamatergic dysfunction in BD. Aberrant excitatory neurotransmission via the N-methyl-D-aspartate (NMDA) receptor contributes to impaired neuroplasticity, frontolimbic dysregulation, and affective instability. Functional imaging studies have revealed hypoactivity in the right ventromedial prefrontal cortex and hyperactivity in the left amygdala, striatum, and anterior cingulate cortex, suggesting lateralized network dysfunction that underlies emotional dysregulation (2, 3). These network alterations may interact with molecular abnormalities, such as reduced myelination and aberrant synaptic pruning, to sustain mood dysregulation (4).

At the molecular level, elevated plasma glutamate and glycine levels have been observed during manic episodes (5), indicating NMDA receptor overactivation. Moreover, alterations in NMDA receptor subunits (NR1, NR2A, NR2B) and regional receptor density have been identified in frontolimbic structures (6). Such findings reinforce glutamatergic imbalance as a central mechanism in BD pathophysiology.

In this context, ketamine—a non-competitive NMDA receptor antagonist—has emerged as a novel and promising treatment for bipolar depression. Administered at sub-anesthetic doses (0.5 mg/kg intravenously over 40 minutes), ketamine produces a rapid and robust antidepressant response, often within hours of infusion (7, 8). Beyond mood improvement, benefits include reductions in anhedonia, suicidal ideation, and anxiety, which are often refractory to conventional therapy.

Recent studies have expanded this paradigm to esketamine, the S-enantiomer of ketamine, which exhibits similar or superior antidepressant efficacy with a more favorable side-effect profile. Evidence from naturalistic and multicentric investigations demonstrates that esketamine is both effective and well-tolerated in patients with treatment-resistant bipolar depression, without evidence of a significant switch risk (9). These findings highlight NMDA receptor modulation as a mechanistically innovative and clinically relevant approach in BD management, warranting systematic evaluation of efficacy, safety, and affective stability.

## Methods

This review was conducted as a structured, narrative synthesis of the available literature on the clinical use of ketamine and esketamine in bipolar depression. The primary focus was to evaluate antidepressant efficacy, safety, and the risk of treatment-emergent affective switching.

A comprehensive literature search was performed using PubMed, Scopus, and Web of Science databases, covering studies published between January 2000 and October 2025. Search terms included combinations of “bipolar disorder,” “bipolar depression,” “treatment-resistant bipolar depression,” “ketamine,” “esketamine,” “NMDA receptor antagonists,” “rapid-acting antidepressants,” and “affective switch.” Only peer-reviewed articles published in English were considered.

Eligible studies included randomized controlled trials (RCTs), open-label or naturalistic clinical studies, systematic reviews, meta-analyses, and preclinical investigations relevant to the mechanism or therapeutic use of ketamine and esketamine in BD. Observational and retrospective studies were included when they provided unique insights into safety, tolerability, or switch risk.

Studies were selected according to the following criteria:

1. Adult participants (aged 18 years or older) diagnosed with bipolar disorder type I or II according to DSM-IV, DSM-5, or ICD-10 criteria.
2. Intervention with ketamine (racemic or R-enantiomer) or esketamine (S-enantiomer) administered via intravenous or intranasal routes.
3. Outcomes including antidepressant efficacy (e.g., reduction in Montgomery–Åsberg Depression Rating Scale [MADRS] or Hamilton Depression Rating Scale [HAM-D] scores), time to response, remission rates, and adverse events.
4. Reporting of treatment-emergent mania, hypomania, or affective switching.

Data were extracted from each study regarding design, sample characteristics, intervention protocol, concomitant medications, and clinical outcomes. Given the limited number of RCTs specifically targeting bipolar populations, a narrative synthesis approach was adopted. Quantitative data were summarized when available, and findings were compared across trials and reviews to highlight consistent trends in antidepressant response and switch risk.

Particular emphasis was placed on:

- Controlled trials demonstrating rapid-onset antidepressant effects of ketamine (7, 8),
- Meta-analytic and systematic data contextualizing efficacy across treatment-resistant bipolar depression (8), and
- Naturalistic and real-world data on esketamine safety and affective stability (9).

Reference lists of all included studies were manually screened to identify additional relevant publications. Articles focusing exclusively on unipolar depression were included only when providing mechanistic or comparative insights relevant to bipolar depression.

## Results

### Clinical efficacy of ketamine in bipolar depression

Multiple clinical trials have demonstrated that low-dose intravenous ketamine produces rapid and robust antidepressant effects in patients with bipolar depression, particularly in those resistant to standard pharmacotherapy. In the pivotal randomized, double-blind, placebo-controlled crossover trial by Diazgranados et al. (7), patients maintained on therapeutic levels of lithium or valproate received a single intravenous infusion of ketamine hydrochloride (0.5 mg/kg). Depressive symptoms improved significantly within 40 minutes, and the effect persisted for up to three days. The response rate exceeded 70%, and adverse effects were mild and transient, with no sustained manic symptoms reported.

These findings were later supported by Zarate et al. and integrated into a meta-analysis by Fornaro et al. (8), which synthesized 17 randomized controlled trials (n = 928). In patients with treatment-resistant bipolar depression (TRBD), the odds of achieving a clinically meaningful response at 24 hours post-infusion were more than tenfold higher compared to placebo (OR = 10.68; 95% CI = 2.14–53.27). The pooled dropout rate across ketamine trials was 21%, indicating reasonable acceptability in this population.

Importantly, across all ketamine studies involving bipolar cohorts, the incidence of treatment-emergent mania or hypomania was low. Only isolated cases of transient mood elevation were observed, typically resolving spontaneously or within hours. These findings support the notion that ketamine’s rapid antidepressant mechanism is distinct from conventional monoaminergic antidepressants, which are more prone to trigger manic switching.

### Esketamine in treatment-resistant bipolar depression

While most controlled studies have focused on racemic ketamine, emerging evidence supports the efficacy and safety of esketamine, the S-enantiomer, in bipolar depression. The multicentric, real-world REAL-ESK study by Martinotti et al. (9) compared outcomes in patients with treatment-resistant unipolar and bipolar depression treated with intranasal esketamine. Among the 35 subjects with bipolar TRD, significant reductions were observed in MADRS, HAM-D, and HAM-A scores at one and three months of follow-up.

Response and remission rates were comparable between unipolar and bipolar groups, indicating that the antidepressant efficacy of esketamine extends to the bipolar spectrum. Importantly, no cases of manic or hypomanic switch were observed, and the treatment was generally well-tolerated. Esketamine also demonstrated a stronger anxiolytic effect in bipolar patients, suggesting potential advantages in addressing comorbid anxiety symptoms.

These real-world findings align with clinical observations from the SUSTAIN-2 study and other phase III trials in unipolar treatment-resistant depression, none of which reported affective switching (10, 11). Collectively, these data suggest that esketamine maintains the rapid antidepressant and antisuicidal effects of ketamine, with a favorable safety profile and minimal risk of mood destabilization.

### Adjunctive and combination treatments

Ketamine has also been investigated as an adjunctive agent to enhance response in other interventions for bipolar depression. Studies combining ketamine with electroconvulsive therapy (ECT) have reported additive benefits, including reduced cognitive side effects and faster recovery of depressive symptoms (12, 13).

Furthermore, evidence suggests that concurrent mood stabilizer therapy—most commonly lithium or valproate—may mitigate the risk of treatment-emergent mania without attenuating the antidepressant effect. This combination appears to maintain affective stability while allowing for the rapid-onset benefits of NMDA receptor blockade (8, 14).

### Summary of findings

Overall, the available evidence consistently demonstrates that both ketamine and esketamine provide significant, rapid, and clinically meaningful antidepressant effects in patients with bipolar depression. Across controlled and naturalistic settings, the risk of mood switching remains minimal, especially when these agents are used adjunctively with mood stabilizers. The therapeutic benefits typically emerge within hours to days and may extend beyond core depressive symptoms to include improvements in anhedonia, anxiety, and suicidal ideation.

## Discussion

The present synthesis highlights the growing body of evidence supporting the use of NMDA receptor antagonists, particularly ketamine and its S-enantiomer esketamine, as rapid-acting antidepressant agents in bipolar depression. These compounds represent a significant paradigm shift in the treatment of affective disorders by targeting glutamatergic dysregulation rather than the traditional monoaminergic pathways.

### Mechanistic considerations

The rapid onset of antidepressant effects observed with ketamine and esketamine contrasts sharply with the delayed efficacy of conventional antidepressants. Mechanistically, sub-anesthetic NMDA receptor blockade enhances synaptic glutamate release, which in turn activates AMPA receptors, upregulates brain-derived neurotrophic factor (BDNF), and stimulates downstream signaling cascades, including the mammalian target of rapamycin (mTOR) pathway (15, 16). This cascade promotes synaptic plasticity and neurogenesis, processes that are disrupted in mood disorders.

Recent evidence suggests that ketamine’s antidepressant mechanism extends beyond NMDA receptor antagonism, involving complex cross-talk with other neurotransmitter systems. In a large randomized controlled trial, Rengasamy et al. (17) found no significant relationships between ketamine treatment, peripheral neurotrophic or inflammatory markers (e.g., BDNF, IL-6, TNFα), and clinical response in patients with treatment-resistant depression, suggesting that peripheral biomarkers do not capture the core central mechanisms of ketamine’s action. Instead, they proposed that central neuroplastic and synaptic mechanisms likely underlie the rapid antidepressant effects.

Complementary experimental evidence from Rizzo et al. (18) demonstrated that esketamine acts via a dual mechanism involving both NMDA receptor antagonism and μ-opioid receptor modulation. In mice, esketamine enhanced dopaminergic tone in the nucleus accumbens by prolonging dopamine clearance while simultaneously decreasing glutamatergic activity—a pattern partially reversed by naloxone. These findings indicate that opioid receptor activation contributes to the modulation of dopaminergic and glutamatergic neurotransmission, thereby supporting a model in which NMDA blockade, opioid signaling, and dopaminergic balance jointly produce rapid antidepressant effects. Such multimodal interactions may also explain the anxiolytic and anti-anhedonic properties of esketamine and its favorable compatibility with concurrent mood stabilizer therapy.

From a clinical perspective, these convergent findings suggest that ketamine’s efficacy likely depends on an integrated neurochemical network involving glutamatergic, opioid, and dopaminergic pathways, rather than on NMDA antagonism alone. This integrative view provides a neurobiological rationale for the compatibility of NMDA modulators with mood stabilizers, which also influence glutamate transmission and neurotrophic signaling. The opioid-mediated component, while potentially enhancing antidepressant efficacy, warrants careful monitoring due to possible reinforcement or dependence-related effects in vulnerable populations.

The antidepressant effect of NMDA modulation is also associated with functional normalization within frontolimbic circuits. Neuroimaging studies demonstrate that ketamine restores connectivity between the prefrontal cortex and limbic structures, facilitating improved emotional regulation and cognitive control (12). Moreover, esketamine may exert stereoselective advantages, with preclinical data suggesting stronger antidepressant potency and reduced psychotomimetic effects compared with the R-enantiomer or racemic formulation (19).

### Clinical efficacy and affective stability

Across controlled and real-world studies, both racemic ketamine and esketamine demonstrate substantial antidepressant efficacy in bipolar depression, including in treatment-resistant populations. The magnitude of effect, as indicated by meta-analytic evidence, is comparable to or exceeds that observed in unipolar depression (8). Crucially, the concern of treatment-emergent mania—a major limitation of traditional antidepressants—appears markedly reduced.

In the landmark RCT by Diazgranados et al. (7), only one case of transient mood elevation occurred during ketamine treatment, which resolved spontaneously. Similarly, in the REAL-ESK study by Martinotti et al. (9), no manic or hypomanic episodes were reported among bipolar TRD patients treated with intranasal esketamine over three months of follow-up. These findings suggest that the risk of switching is minimal, especially when ketamine or esketamine is administered in combination with mood stabilizers.

Furthermore, the concurrent use of lithium or valproate appears to enhance affective stability without attenuating antidepressant efficacy (8). This observation reinforces the hypothesis that NMDA receptor antagonism may achieve antidepressant effects through neuroplastic rather than mood-destabilizing mechanisms.

### Safety, tolerability, and practical implications

Both ketamine and esketamine are generally well tolerated when administered under clinical supervision. The most frequently reported adverse effects—transient dissociation, dizziness, or mild increases in blood pressure—are short-lived and self-limiting. Compared with intravenous racemic ketamine, esketamine offers a more convenient route of administration via intranasal spray, making it suitable for outpatient treatment and long-term maintenance under controlled protocols.

However, several limitations remain. Long-term data on repeated or maintenance dosing in bipolar populations are scarce. Most studies to date have focused on short-term efficacy, and the durability of antidepressant response beyond several weeks remains to be established. Additionally, questions persist regarding the optimal dosing frequency, potential for tolerance, and long-term neurocognitive or cardiovascular effects.

### Integration within bipolar disorder management

Given its rapid antidepressant action and low switch risk, esketamine represents a valuable adjunctive treatment option for patients with bipolar depression who have failed conventional therapies. Its use aligns with the broader trend in psychopharmacology toward personalized, mechanism-based approaches that address specific neurobiological abnormalities. NMDA receptor modulation may be particularly beneficial in cases marked by glutamatergic imbalance, impaired neuroplasticity, or treatment resistance.

Nevertheless, clinical integration requires careful consideration. Esketamine should be administered as part of a comprehensive treatment plan that includes ongoing mood stabilizer therapy, close monitoring for affective changes, and structured follow-up to ensure safety and efficacy. Further research should focus on identifying predictive biomarkers of response, exploring maintenance strategies, and optimizing combination regimens with existing mood-stabilizing and psychotherapeutic interventions.
